# Driving Intention Recognition of Surrounding Vehicles Based on a Time-Sequenced Weights Hidden Markov Model for Autonomous Driving

**DOI:** 10.3390/s23218761

**Published:** 2023-10-27

**Authors:** Pujun Liu, Ting Qu, Huihua Gao, Xun Gong

**Affiliations:** 1 National Key Laboratory of Automotive Chassis Integration and Bionics, Changchun 130022, China; liupj21@mails.jlu.edu.cn (P.L.); quting@jlu.edu.cn (T.Q.); gongxun@jlu.edu.cn (X.G.); 2 School of Artificial Intelligence, Jilin University, Changchun 130025, China

**Keywords:** advanced driver assistance system, autonomous driving, driving intention recognition, time-sequenced weights hidden Markov model, lane changing

## Abstract

Accurate perception, especially situational awareness, is central to the evolution of autonomous driving. This necessitates understanding both the traffic conditions and driving intentions of surrounding vehicles. Given the unobservable nature of driving intentions, the hidden Markov model (HMM) has emerged as a popular tool for intention recognition, owing to its ability to relate observable and hidden variables. However, HMM does not account for the inconsistencies present in time series data, which are crucial for intention recognition. Specifically, HMM overlooks the fact that recent observations offer more reliable insights into a vehicle’s driving intention. To address the aforementioned limitations, we introduce a time-sequenced weights hidden Markov model (TSWHMM). This model amplifies the significance of recent observations in recognition by integrating a discount factor during the observation sequence probability computation, making it more aligned with practical requirements. Regarding the model’s input, in addition to easily accessible states of a target vehicle, such as lateral speed and heading angle, we also introduced lane hazard factors that reflect collision risks to capture the traffic environment information surrounding the vehicle. Experiments on the *HighD* dataset show that TSWHMM achieves recognition accuracies of 94.9% and 93.4% for left and right lane changes, surpassing both HMM and recurrent neural networks (RNN). Moreover, TSWHMM recognizes lane-changing intentions earlier than its counterparts. In tests involving more complex roundabout scenarios, TSWHMM achieves an accuracy of 87.3% and can recognize vehicles’ intentions to exit the roundabout 2.09 s in advance.

## 1. Introduction

While the advanced driver assistance system (ADAS) has progressed significantly [1,2], challenges in perception modules still impede the broader evolution of autonomous driving. These modules, responsible for gathering traffic data and predicting scenarios [3], are crucial for ensuring autonomous vehicles effectively respond to dynamic and potentially hazardous situations. Given the pivotal role of perception modules, timely recognition of the driving intentions of surrounding vehicles becomes paramount, directly influencing the subsequent decision-making process of autonomous vehicles. However, existing driving intention recognition algorithms often underperform, leading to reduced safety and efficiency in ADAS. Thus, developing an algorithm that can accurately and promptly recognize the driving intention of vehicles becomes crucial to enhance automotive intelligence.

On highways, driving intentions can be broadly categorized into lane changing left (LCL), lane changing right (LCR), and lane keeping (LK). Statistics reported in [4] show that 75% of lane changing (LC) crashes are due to the driver misunderstanding the intentions of surrounding vehicles, highlighting the importance of accurate and timely detection of these intentions for driver safety. In theory, properly using turn signals helps drivers accurately discern LC intentions. However, in practice, many drivers neglect to activate their turn signals before a lane change for various reasons. A study conducted in Beijing, China, revealed that less than 40% of drivers used their turn signals during LC [5]. Similarly, research from Ohio, USA, indicated that 48.35% of drivers did not adhere to the turn signal regulations during LC [6]. Furthermore, even when drivers are aware of the need to use their turn signals, variations among drivers combined with unpredictable driving conditions, lead to significant instability in the time interval between signaling and actual lane changing. Thus, solely relying on turn signals to gauge another vehicle’s LC intention is unreliable. To solve this problem, researchers are exploring other indicators, like vehicle speed and steering wheel angle, for a clearer understanding of drivers’ LC intentions.

**Figure 1:**
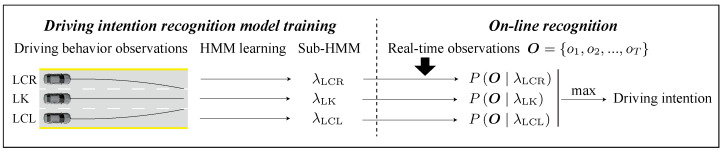
Basic framework of driving intention recognition (HMM-based). *T* stands for the current moment.

**Figure 2:**
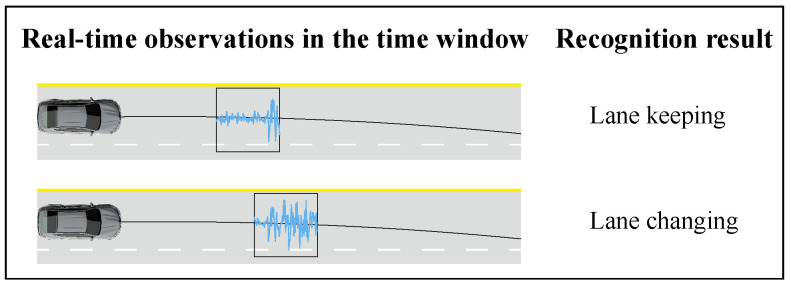
Hysteresis of HMM-based intention recognition models. In the early stage of lane changing, most of the data within the time window is LK data, so the recognition result is LK. When LC data dominates, it is recognized as LC.

In driving scenarios, actions and intentions evolve sequentially, with factors like vehicle speed, steering angle, and turn signal usage forming a data “time series”. The HMM is particularly effective in analyzing and processing this type of data. A basic framework of the HMM-based driving intention recognition model is shown in Figure 1, where the corresponding HMMs λ are trained on different sets of driving behavior observations (LCR, LK, and LCL). For online recognition, the current real-time observations are input to the three models for matching, and the model with the highest likelihood is the driver’s driving intention at the current moment. Recognizing driving intention early is crucial, especially in high-speed situations, where even a millisecond’s delay can lead to severe consequences. The HMM-based intention recognition model considers the behavior model with the highest likelihood from the overall observations, which makes the LC behavior observation data necessary to occupy most of the real-time observations before it can be correctly recognized by the model (Figure 2). This prevents the model from identifying the correct intention in the early stages of lane changing. In reality, not all observations are of equal significance since the most crucial piece of information is the driver’s current intention. The closer the data is to the current moment, the greater its value. Thus, it is essential to assign different weights to observations at each timestep based on their proximity to the current moment.

In this paper, we introduce the TSWHMM to enhance the model’s effectiveness in the early stages of lane changing. During the intention recognition process, TSWHMM adaptively adjusts the weights of observations at each timestep, placing greater emphasis on observations closer to the current moment. We conducted training tests using the *HighD* dataset from highway scenarios [7] and analyzed the results in conjunction with the vehicle’s driving trajectory. Our experiments demonstrate that TSWHMM achieves recognition accuracies of 94.9% and 93.4% for LCL and LCR, respectively, outperforming both HMM and RNN. Furthermore, in the early stages of lane changing, TSWHMM exhibits superior performance, recognizing the lane-changing intention of vehicles 4.1 s before they cross the lane markings, on average. Beyond highway scenarios, we extended our tests to roundabout environments to address the concerns regarding the model’s generalization. The results affirm that TSWHMM maintains its efficacy, suggesting its robustness and adaptability across diverse driving contexts.

## 2. Related Work

Recognizing driving intentions involves interpreting the driver’s specific intentions from observed data. Consequently, much work has examined various discriminative models, such as support vector machine (SVM) [8,9,10], neural networks (NNs) [11,12], *k*-nearest neighbors (*k*-NN) [13], and so on. Although these discriminative models excel in many tasks, they are primarily designed for static data and may struggle to capture temporal relationships within the data. Given that driving behaviors are sequential, with each action influenced by its predecessors [14], capturing temporal aspects is paramount. Thus, discriminative models failing to grasp the temporal nuances within data might misinterpret a driver’s genuine intentions. In contrast, sequential models, such as RNN [15,16,17], dynamic Bayesian networks (DBN) [18,19], and HMM, are specifically designed to capture and process temporal dependencies in data. For tasks like driving intention recognition, these sequential models may offer more accurate and reliable results [20]. In [21], researchers evaluated the performance of the dynamic time warping (DTW) feature combined with *k*-NN in comparison to the sequence prediction using a combined HMM for lane-change detection. The results indicated the marked superiority of the HMM-based approach, suggesting the robustness of sequential models in capturing driving behaviors.

However, each sequential model presents its own unique set of challenges [22,23]. For example, the RNN demands considerable computational resources during training because of the complexities associated with backpropagation through time [24]. While it is efficient for real-time predictions, it may falter when adapting to long sequences and can be plagued by issues like vanishing and exploding gradients [25,26]. The DBN, though versatile, has slower real-time inference [27], making it less suitable for immediate driving scenarios. Its intricate modeling can introduce unnecessary complexity [28], and, when compared to HMM, it might require more data for effective training [29]. Notably, the HMM model adeptly captures the probabilistic relationship between observable actions and hidden intentions [30,31], fitting the nature of intention recognition. Moreover, it is computationally efficient in both training and real-time inference [32], avoiding pitfalls like the vanishing gradient. Its ability to integrate various observations, from vehicle speed to steering angle, and handle sequences of varying lengths ensures a comprehensive and adaptable approach to intention recognition.

Worrall et al. [33] were the first to treat the LC process as a stochastic process with Markovian properties. They developed a corresponding LC model calibrated using data from a 6-lane stretch of highway in Chicago. The authors of [34] regarded the LC process as a series of linear actions, including checking the mirrors, turning on the turn signal, initiating the turn, and aligning the vehicle in the target lane. As a result, they selected left-right HMM for recognizing the driver’s early LC intention. The authors of [35,36] proposed a driver behavior framework for intersections, which couples the driver behavior and vehicle state as a hybrid-state system (HSS). The driver’s decision is represented as a high-level discrete state system, while the vehicle state is represented as a low-level continuous state system. The framework employs an HMM to estimate the driver’s behavior from filtered continuous observations. In [37], a human-like lane changing intention understanding model (HLCIUM) was proposed, which simulates the selective attention mechanism of the human visual system, focuses attention on the relevant vehicle based on salience schemes, and then recognizes the driving intention through HMM.

The existing driving intention recognition models retain the inherent mathematical structure of the HMM without distinguishing the importance of time-varying observations. Consequently, there remains significant room for improvement in their recognition capabilities. Given the intricacies of intention recognition, where recent observations often hold more relevance, it is evident that the traditional HMM structure requires modifications to better capture these nuances. However, no relevant research has been conducted on this aspect yet.

The main contributions of this paper are as follows:

- We propose TSWHMM, a novel algorithm that accounts for the varying significance of observations in intention recognition. By integrating a discount factor into the calculation of the observation sequence probability, it amplifies the influence of recent observations, enhancing the model’s recognition capability. Crucially, this algorithm can be easily generalized to other HMM-based intention recognition methods without substantially increasing complexity.
- Lane hazard factors are designed as part of the observation variable to uncover hidden information about the surrounding traffic environment and to improve the situational awareness of the driving intention recognition model. This significantly improves the recognition of the model.
- Tests were conducted in both highway and roundabout scenarios to explore the impact of the discount factor on the model, offering a deeper understanding of the workings of TSWHMM. Comparative experiments with HMM and RNN further revealed that TSWHMM boasts superior intention recognition accuracy and exhibits enhanced performance during the early stages of lane-changing.

## 3. Theoretical Description of TSWHMM

### 3.1. Driving Intention Recognition Process of TSWHMM

**Figure 3:**
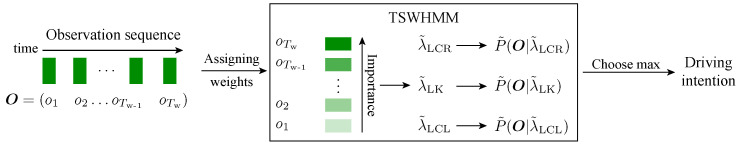
Framework of TSWHMM-based driving intention recognition system. $T_{w}$ is the length of the observation sequence.

The process of driving intention recognition through TSWHMM is shown in Figure 3. The input observation sequence $O=(o_{1},o_{2},\ldots ,o_{T_{w}})$ is first determined based on the size of the time window $T_{w}$, where $o_{T_{w}}$ represents the current momentary observation data. After receiving the observation sequence, TSWHMM assigns weight coefficients in temporal order, emphasizing data closer to the current moment as more important. The trained behavioral models, namely ${\tilde{\lambda }}_{\mathrm{LCR}}$, ${\tilde{\lambda }}_{\mathrm{LK}}$, and ${\tilde{\lambda }}_{\mathrm{LCL}}$, then compute the observation sequence probability with temporal weights, denoted as $\tilde{P}\left(O|\tilde{\lambda }\right)$, respectively. Finally, the driving intention of the model with the highest probability is recognized as the current moment’s result. If there are two or more models with the highest probability, the result from the previous moment is chosen as the current moment’s result. This process is repeated for each subsequent moment until completion.

The observation data $o_{t}$ comprises the variables listed in Table 1. Among them, Δy, $v_{y}$, $a_{y}$, and θ are readily accessible vehicle state variables, while the lane hazard factor ρ quantifies the level of danger for the target vehicle in each lane. They are mathematically defined as follows:

(1) $$\rho_{i}=\left\{\begin{array}{ccc} \sum_{A}TTC^{-1} & ,\; & \mathrm{if}\;\rho_{i}<a\\ a & ,\; & \mathrm{otherwise}. \end{array}\right.$$

(2) $$TTC^{-1}=\left\{\begin{array}{ccc} 0 & ,\; & \mathrm{if}\;TTC^{-1}\leq 0\\ \frac{v_{\mathrm{obe}\_x}-v_{\mathrm{other}\_x}}{x_{\mathrm{other}}-x_{\mathrm{obe}}} & ,\; & \mathrm{otherwise}. \end{array}\right.$$

where i=leftlane,rightlane,currentlane. The meanings of the parameters in (1) and (2) are shown in Table 2. A road-based reference coordinate system is established with the road’s centerline as the x-axis and its vertical direction as the y-axis. As indicated by (2), when the $TTC^{-1}$ is negative, it signifies the absence of any risk of collision between two vehicles. To facilitate model training, all negative values are uniformly assigned a value of 0. Additionally, since the $TTC^{-1}$ values may tend to infinity, which again may adversely affect the training of the model, the upper limit of the parameter ρ is restricted to a finite value to indicate the absolute degree of danger. If there is no lane on one side of the target vehicle, the corresponding ρ can be set to *a* to indicate absolute danger (no possibility of changing lanes to that side).

It should be noted that the lane hazard factor, as a newly defined observation variable, is not exclusive to TSWHMM. Any model can incorporate this variable as one of its inputs. In subsequent comparative experiments, this variable is incorporated into the input sets, except in the case of the roundabout experiments.

### 3.2. Difference between HMM and TSWHMM

The parameters of TSWHMM are denoted as $\tilde{\lambda }=\left(\pi ,A,\phi ,\gamma \right)$. Compared to the HMM parameters λ=(π,A,ϕ), TSWHMM includes an additional discount factor γ to adjust the temporal weights of the observation sequence. The algorithm employed to estimate the parameters π, A and ϕ aligns with that of the HMM. However, γ serves as a model hyperparameter without a direct estimation formula, and must be determined based on either practical needs or optimization benchmarks (e.g., the accuracy of the test set). In this study, an optimization search was conducted over a series of discrete values for γ to determine its optimal value.

Instead of calculating the observation sequence probability P(O|λ), TSWHMM computes the observation sequence probability $\tilde{P}\left(O|\tilde{\lambda }\right)$ that considers the temporal weights, which are controlled by γ. This is the major difference between HMM and TSWHMM.

### 3.3. Principle of TSWHMM

In this section, the formula of TSWHMM for calculating $\tilde{P}\left(O|\tilde{\lambda }\right)$ will be derived from HMM, and the advantages of TSWHMM in the intention recognition problem will be illustrated at the same time.

**Figure 4:**
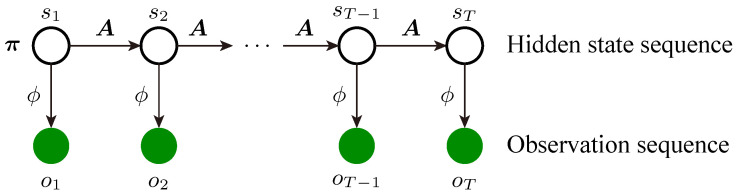
Graphical structure of HMM.

The graphical structure of HMM for generating the observation sequence is shown in Figure 4. First, the model generates the initial state $s_{1}$ controlled by $\pi =(\pi_{1},\pi_{2},\ldots ,\pi_{N})$, where *N* is the number of states, and $\pi_{i}=P\left(s_{1}=i|\pi \right)$. Then, the complete hidden state sequence $S=(s_{1},s_{2},\ldots ,s_{T})$ is generated according to the state transition matrix $A={\left[a_{ij}\right]}_{N\times N}$, where $a_{ij}=P\left(s_{t}=j|s_{t-1}=i,A\right)$, i,j=1,2,…,N. Finally, the observation sequence $O=(o_{1},o_{2},\ldots ,o_{T})$ is determined based on S and ϕ. In this paper, Gaussian mixture models (GMM) are used to fit the observation probability:

(3) $$\begin{array}{cc} b_{i}\left(o_{t}\right) & =P(o_{t}|s_{t}=i,\phi )\\ \; & =\sum_{m=1}^{M}c_{im}N\left(o_{t}|\mu_{im},\Sigma_{im}\right), \end{array}$$

where *M* is the number of Gaussian components; $c_{im}$ is the mixing coefficient of the *m*th Gaussian component under state *i*, satisfying the constraint $\sum_{m=1}^{M}c_{im}=1$. So, we have ϕ=(c,μ,Σ).

Therefore, given the HMM parameters λ=(π,A,ϕ) and observation sequence O, the observation sequence probability P(O|λ) can be written as:

(4) $$P\left(O|\lambda \right)=\sum_{S}P\left(O,S|\lambda \right),$$

which means that P(O|λ) is equal to the sum of P(O,S|λ) over all possible hidden state sequences S.

Assuming that one possible hidden state sequence is $S^{\prime }=\left(s_{1}^{\prime },s_{2}^{\prime },\ldots ,s_{T}^{\prime }\right)$, the joint probability distribution of the observation sequence O and the hidden state sequence $S^{\prime }$ under the given model λ is

(5) $$\begin{array}{cc} P(O,S^{\prime }|\lambda )= & P\left(s_{1}^{\prime }|\pi \right)P\left(o_{1}|s_{1}^{\prime },\phi \right)\\ \; & \times \prod_{t=2}^{T}P\left(s_{t}^{\prime }|s_{t-1}^{\prime },A\right)P\left(o_{t}|s_{t}^{\prime },\phi \right). \end{array}$$

If we set:

(6) $$P_{O,S^{\prime }}\left(1\right)=P\left(s_{1}^{\prime }|\pi \right)P\left(o_{1}|s_{1}^{\prime },\phi \right)$$

(7) $$P_{O,S^{\prime }}\left(t\right)=P\left(s_{t}^{\prime }|s_{t-1}^{\prime },A\right)P\left(o_{t}|s_{t}^{\prime },\phi \right),\;\;\;\;t=2,\ldots ,T$$

and combining (5)–(7), we get:

(8) $$P\left(O,S^{\prime }|\lambda \right)=\prod_{t=1}^{T}P_{O,S^{\prime }}\left(t\right),$$

taking the logarithm:

(9) $$\ln P(O,S^{\prime }|\lambda )=\sum_{t=1}^{T}\ln P_{O,S^{\prime }}\left(t\right),$$

where $P_{O,S^{\prime }}\left(1\right)$ denotes the probability that at moment t=1 the state is $s_{1}^{\prime }$ and the observation is $o_{1}$; $P_{O,S^{\prime }}\left(t\right)\left(t\neq 1\right)$ denotes the probability that given $s_{t-1}^{\prime }$, the state at moment *t* is $s_{t}^{\prime }$ and the observation is $o_{t}$.

Equation (9) demonstrates that, under specific constraints, the logarithm of the joint probability distribution of O and $S^{\prime }$, $\ln P(O,S^{\prime }|\lambda )$, can be obtained by summing the logarithm of the generation probability of the observation, $o_{t}$, at each time step *t*. However, in the context of intention recognition, the importance of observations increases as they get closer to the current time. The closer the observations are to the current moment *T*, the more accurately they reflect the driving intention of the vehicle. Therefore, the importance of $P_{O,S^{\prime }}\left(t\right)$ varies. To better address the actual problem, the weight of observations closer to the current moment should be increased by adjusting their significance within the sequence.

Considering the input as a time-ordered sequence, we assign an exponentially time-dependent weight factor $\gamma^{T-t}$ to $\ln P_{O,S^{\prime }}\left(t\right)$:

(10) $$\ln\tilde{P}\left(O,S^{\prime }|\tilde{\lambda }\right)=\sum_{t=1}^{T}\gamma^{T-t}\ln P_{O,S^{\prime }}\left(t\right),$$

where γ is the discount factor and falls within the range 0<γ≤1; $\tilde{P}\left(O,S^{\prime }|\tilde{\lambda }\right)$ is the joint probability distribution considering the temporal weights. This approach is inspired by the discount factor utilized in reinforcement learning. The factor γ ensures that the weight of $\ln P_{O,S^{\prime }}\left(t\right)$ of the current moment remains 1, while the weight assigned to earlier moments increases exponentially as we approach the current moment. With this method, the likelihood of the model representing the true driving intention is significantly increased, thus improving the final result.

While this conclusion is based on a single hidden state sequence $S^{\prime }$, it is evident from (4) that P(O|λ) is the sum of $P(O,S^{\prime }|\lambda )$ across all possible hidden state sequences. Hence, the conclusion remains valid for P(O|λ). Therefore, the probability of an observation sequence considering the temporal weights, is:

(11) $$\tilde{P}\left(O|\tilde{\lambda }\right)=\sum_{S}\tilde{P}\left(O,S|\tilde{\lambda }\right).$$

When the $\tilde{P}\left(O|\tilde{\lambda }\right)$ cannot be calculated directly from (11), we refer to the forward algorithm [38] to solve it. Since (10) is a power operation on the joint distribution probabilities at each time step (note that the discount factor γ is multiplied by the joint probability after taking the logarithm) and operates consistently for all hidden state paths S (γ is only related to *t*), it is straightforward to write the recursive equation for $\tilde{P}\left(O|\tilde{\lambda }\right)$ with the help of a forward algorithm as follows:

1. Initial value:
  (12) $${\tilde{\alpha }}_{1}\left(i\right)={\left[\pi_{i}b_{i}\left(o_{1}\right)\right]}^{\gamma^{T-1}},\;i=1,\cdots ,N$$
2. Recursion:
  (13) $${\tilde{\alpha }}_{t}\left(i\right)=\sum_{j=1}^{N}{\tilde{\alpha }}_{t-1}\left(j\right){\left[a_{ji}b_{i}\left(o_{t}\right)\right]}^{\gamma^{T-t}},$$
  where t=2,3,⋯,T.
3. Termination:
  (14) $$\tilde{P}\left(O|\tilde{\lambda }\right)=\sum_{i=1}^{N}{\tilde{\alpha }}_{T}\left(i\right).$$

It is important to note that when γ=1, the TSWHMM simplifies to a classical HMM. This characteristic allows us to investigate the influence of γ on the intention recognition problem, further highlighting the flexibility of TSWHMM.

## 4. Simulation Preprocessing

All computational experiments and analyses were conducted on a system running Windows 10. The hardware utilized included an Intel(R) Xeon(R) Silver 4114 CPU operating at 2.20 GHz and an NVIDIA GeForce RTX 3080 GPU. For software, MATLAB R2023a was employed throughout the study.

### 4.1. Data Processing

The *HighD* dataset was used to validate the effectiveness of TSWHMM for driving intention recognition. It is a naturally recorded driving dataset obtained through the use of an unmanned aerial vehicle (UAV) on a German highway. The highway stretches over 420 m, and the data were collected at a 25 Hz frequency. The dataset provides a valuable third-party perspective, detailing vehicle status, lane specifics, and lane-change events. More importantly, it contains 5600 recorded complete lane changes. This information makes the *HighD* dataset an ideal source for validating the performance of algorithms for recognizing the driving intentions of surrounding vehicles.

**Figure 5:**
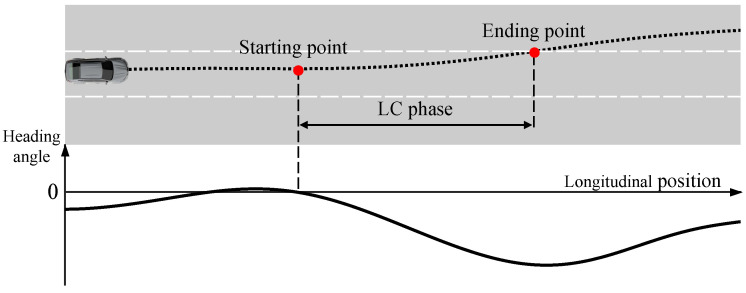
Definition of LC phase.

**Figure 6:**
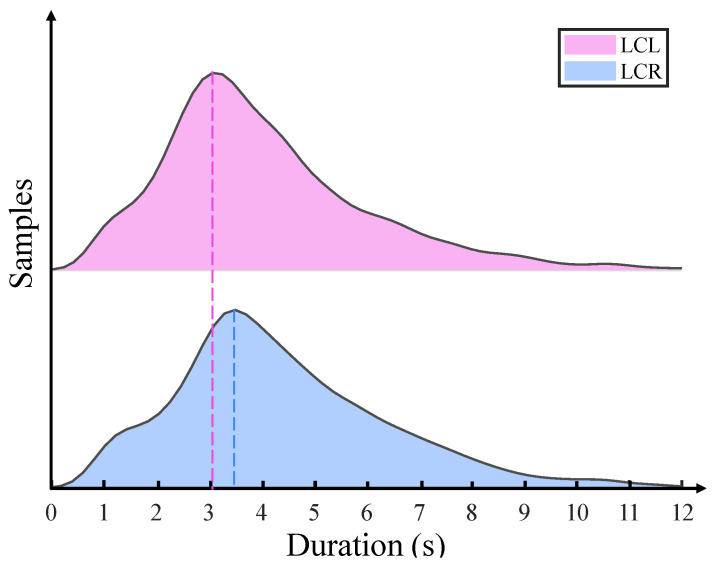
LC phase duration distribution.

The subsequent section details the methodology for extracting LC phase data from the *HighD* dataset. The LC phase lacks definite starting and ending points for lane changes, and manual labeling is usually required. Given the vast amount of data, manual labeling is inefficient, underscoring the need for a clear definition of the lane changing process. We define the LC phase as follows: The moment the last heading angle reaches zero before the vehicle crosses the lane line marks the LC start. The point where the vehicle’s center crosses the lane line denotes the LC end. The duration between these points is the LC phase (Figure 5). Extracting data for the LK phase is easier. It is only necessary to ensure that the sample size is similar to that of the LC phase. Table 3 shows the number of LC phase samples extracted from *HighD* based on the above definition. Figure 6 provides a statistical representation of the duration distribution for the two LC phases. Overall, the duration of both is around 3 s, with the LCL phase being shorter than the LCR phase. This may be because most LCL behaviors aim to overtake or switch to a faster lane. The samples are divided into training and test sets at a 4:1 ratio.

### 4.2. Model Training

For driving intention recognition, we must train three sub-TSWHMMs: ${\tilde{\lambda }}_{\mathrm{LCL}}$, ${\tilde{\lambda }}_{\mathrm{LCR}}$, and ${\tilde{\lambda }}_{\mathrm{LK}}$ (see Figure 3). We estimate the parameters π, A, and ϕ using the learning algorithm of the GMM-HMM, as described in [39]. Table 4 lists the TSWHMM hyperparameters. Some are determined empirically based on data characteristics, while others are set within a specific range for grid search.

**Figure 7:**
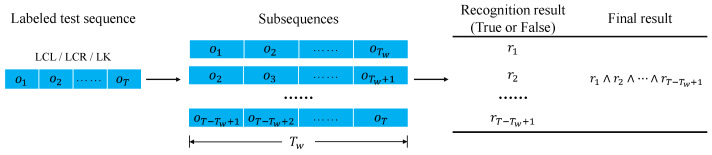
Criteria for whether a test sequence is correctly recognized.

The criteria for determining whether a test sequence is recognized correctly are illustrated in Figure 7. For a test sequence with marked driving behavior, it is first divided into several subsequences based on the time window $T_{w}$. Next, TSWHMM determines the driving intention for each subsequence. If all the recognition results match the label, the test sequence is deemed correctly recognized. Otherwise, it is considered incorrect.

To compare the recognition effects of HMM and TSWHMM, and to highlight the role of the discount factor γ in intention recognition, we will maintain consistent parameters for both models. First, we will determine the optimal HMM parameter set $N_{\mathrm{LCL}},\;N_{\mathrm{LCR}},\;N_{\mathrm{LK}}$. Then, we will perform a grid search for γ in TSWHMM. As shown in Table 5, we present the optimal set of parameters and their corresponding accuracy on the test set.

## 5. Simulation Analysis

### 5.1. Comparsion between TSWHMM and HMM

In this subsection, we compare the accuracy and early lane change recognition performance of HMM and TSWHMM using the test set.

**Figure 8:**
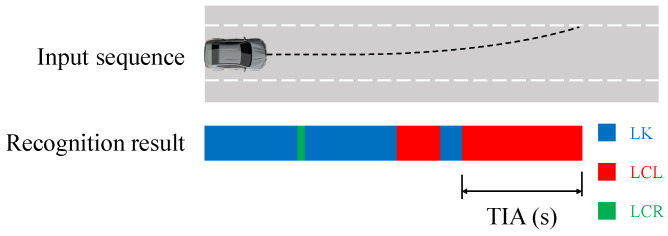
The definition of TIA. The input sequence includes LK and LC phases.

The definition of accuracy is consistent with the previous section (Figure 7). The effectiveness of the model in recognizing the early lane change phase of the vehicle is measured by the time in advance (TIA). TIA is defined as the interval between the moment when the model finally recognizes the intention of the target vehicle as LK and the moment when the target vehicle crosses the lane line (Figure 8). The test set for calculating TIA, which includes both LK and LC phases, differs from the one used for accuracy, as detailed in Table 6. The larger the TIA value, the earlier the model can recognize the vehicle’s intention to change lanes.

**Figure 9:**
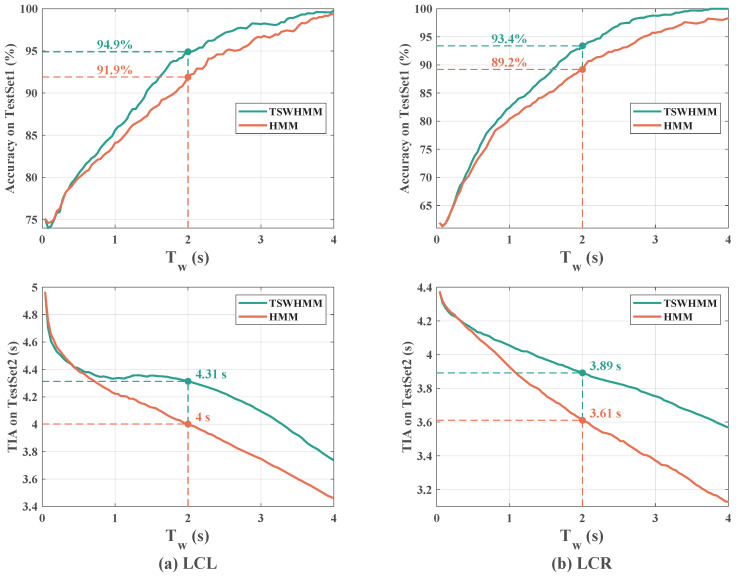
The recognition accuracy and average TIA of TSWHMM and HMM, for LCL and LCR at different $T_{w}$ in TestSet1 and TestSet2.

When using trained HMM or TSWHMM for driving intention recognition, the length of the time window $T_{w}$ significantly impacts both the accuracy and TIA (Figure 9). As $T_{w}$ increases, the accuracy of various HMM-based driving intention recognition algorithms improves. However, this comes at the cost of a significant decrease in TIA, meaning that the trade-off between accuracy and TIA cannot be reconciled. This occurs because the accuracy test set is composed of only one type of driving behavior. The larger the $T_{w}$, the higher the likelihood between the observation sequence and the true behavior model, and, thus, the higher the accuracy. The TIA test set, on the other hand, contains both LK and LC phases, with the LK data entering the time window before the LC data. When the target vehicle starts to change lanes, a majority of the data within the time window has LK features. As $T_{w}$ increases, there is a corresponding increase in the amount of data representing LK features, which decreases the likelihood of recognizing that the target vehicle has started to change lanes, resulting in a decrease in TIA. However, whether it is LCL or LCR, the accuracy and TIA of driving intention recognition with TSWHMM consistently outperform those using the HMM for any value of $T_{w}$. When $T_{w}$ is set to 2 s, TSWHMM achieves 94.9% and 93.4% accuracy for LCL and LCR, respectively, surpassing HMM’s 91.9% and 89.2%. At this setting, TSWHMM can recognize the target vehicle’s intention to change lanes 0.3 s earlier than HMM, on average. This can be attributed to the discount factor γ in TSWHMM—it diminishes the influence of distant data while amplifying the impact of recent data, thereby enhancing the recognition performance.

**Figure 10:**
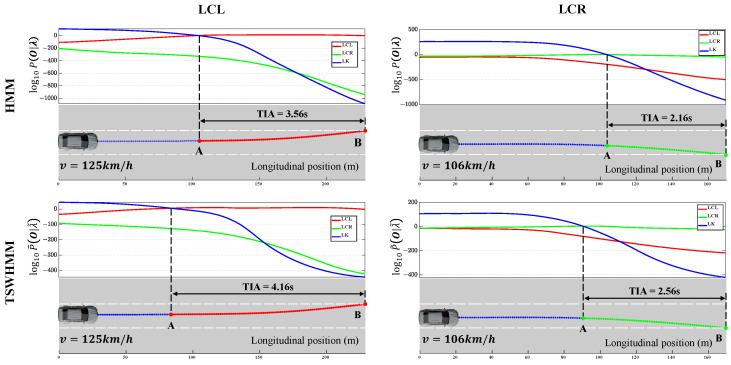
Correspondence between P(O|λ) or $\tilde{P}\left(O|\tilde{\lambda }\right)$ and vehicle trajectory for each behavioral sub-model of HMM and TSWHMM. A and B are starting and ending points of lane-changing, respectively.

Combined vehicle trajectories offer a more intuitive demonstration of driving intention recognition. Figure 10 displays the intention recognition effect of both HMM and TSWHMM on a segment containing LK- and LC-phase driving trajectories. The coordinate domain represents the logarithmic value of P(O|λ) or $\tilde{P}\left(O|\tilde{\lambda }\right)$ of each sub-model of behavior at each sampled point. For each sample point, the recognized intention corresponds to the model with the highest value of P(O|λ) or $\tilde{P}\left(O|\tilde{\lambda }\right)$. As can be seen, the curve of the coordinate domain for the TSWHMM is slightly “shifted” to the left compared to the HMM, resulting in an improvement in the TIA. This provides a clear visual representation of how the observation sequence weights are temporally adjusted by γ, effectively highlighting the superiority of TSWHMM over HMM.

### 5.2. Effect of Discount Factor

In this study, we define the range of the discount factor γ as the interval (0, 1]. To rigorously assess the impact of varying γ values on our intention recognition model, we systematically explored this range, incrementing by 0.01, starting from 0.01 and ending at 1. Notably, as γ decreases, the importance of distant data points diminishes.

**Figure 11:**
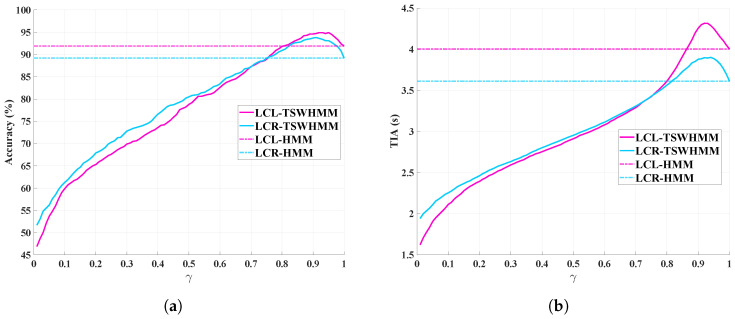
Influence of various γ values on the intention recognition model based on TSWHMM. When γ=1, the model reduces to HMM. (**a**) Accuracy variation in LCL and LCR with different γ values. (**b**) TIA variation in LCL and LCR with different γ values.

Figure 11a,b illustrate the effects of different γ values on model accuracy and TIA, respectively. As mentioned earlier, when γ=1, the TSWHMM model simplifies to a classical HMM. From the figure, it is evident that as γ decreases from 1 and approaches 0 (without reaching it), both the model’s accuracy and TIA initially rise, then decline. With a higher γ, TSWHMM outperforms HMM in all aspects; however, it significantly deteriorates for extremely low γ values. This decline results from the disproportionately low weights given to distant data points, causing the model to overly rely on current data and to overlook the benefits of temporal sequences. When γ is infinitely close to 0, the model would then focuses solely on the current data point. This essentially turns the intention recognition process into a simple data classification using GMM, eliminating the advantages of having a temporal model. Therefore, an appropriate γ in the TSWHMM model not only harnesses the strengths of temporal sequences but also compensates for the inadequacies of HMM in data weight distribution.

### 5.3. Effect of Lane Hazard Factors

To recognize and predict the driving intention of the target vehicle early on, merely relying on its own state is insufficient. The lane hazard factor ρ evaluates each lane’s risk by gauging the potential for a collision between the target vehicle and others. This assists the intention recognition model in making earlier judgments about the target vehicle’s lane change intentions.

**Figure 12:**
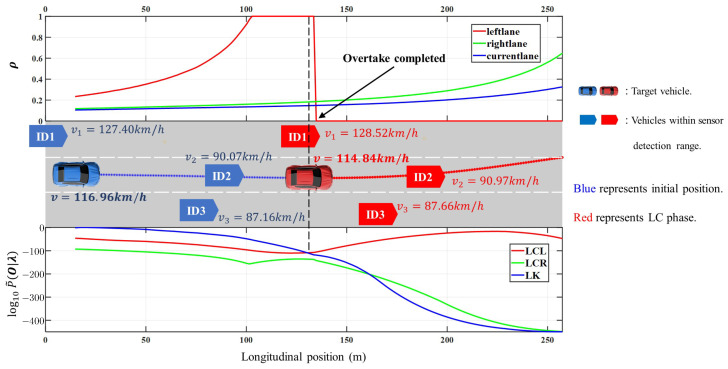
The effect of lane hazards on the intention recognition model. When vehicle ID1 in the left lane is about to overtake the target vehicle, the driving intention recognition model predicts that the target vehicle will make a lane change to the left.

Figure 12 illustrates the assistance provided by ρ to the driving intention recognition model. Initially, the target vehicle is in the central lane with slow-moving vehicles ahead and to its front right, while vehicle ID1 approaches rapidly from the rear left. The value of ρ in the current lane is slowly increasing, indicating the potential collision danger for the target vehicle with the preceding vehicle; therefore, it is inevitable that the target vehicle will eventually change lanes. However, the gradual rise of ρ in the right lane indicates that a slow vehicle has occupied the lane-changing space in the front right, thus the $\tilde{P}\left(O|\tilde{\lambda }\right)$ of the LCR model is the lowest. At the same time, the quick increase in ρ in the left lane indicates that a vehicle is rapidly approaching, making it temporarily infeasible to change to the left lane, so the model recognition result is to continue straight ahead. When the vehicle ID1 on the left approaches the target vehicle, the model anticipates a left lane change intention for the target vehicle, even before any lane change action occurs. This is due to the collision hazard in the current lane and the absence of lane change conditions in the right lane. Consequently, the $\tilde{P}\left(O|\tilde{\lambda }\right)$ of the LCL model becomes the highest, enabling prediction of the driving intention. The results confirm the model’s accuracy, with the lane hazard factor ρ enhancing its performance during the early stages of lane changes.

### 5.4. Comparison with RNN

**Figure 13:**
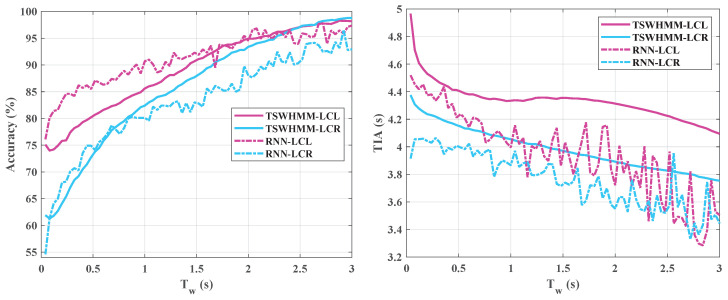
Performance comparison of TSWHMM and RNN for LCL and LCR under varying time windows.

The RNN serves as a prototypical example of sequence models and has been established as a primary technique for driving intention recognition. For a direct comparison between TSWHMM and RNN on this task, we continued using accuracy and TIA as our evaluative metrics. Since both models emphasize temporal sequences, the input sequence length $T_{w}$ will affect their performance. Thus, we examined how these performance metrics varied with changes in sequence length, with the detailed results illustrated in Figure 13.

Both models show similar trends as $T_{w}$ increases—accuracy rises while TIA decreases. Notably, compared to the RNN, the recognition accuracies of TSWHMM for the LCL and LCR intentions are closer together, indicating that TSWHMM achieves a better balance between these two categories. Moreover, as $T_{w}$ increases, TSWHMM’s TIA decline is more gradual than the RNN’s. This suggests that TSWHMM can achieve similar or better accuracy with a smaller TIA compromise than the RNN. Evaluations at $T_{w}=2$ s reveal, as presented in Table 7, that TSWHMM outperforms RNN across the board at this juncture.

### 5.5. Roundabout Scenarios

Recognizing driving intentions in roundabout scenarios is crucial due to the unique navigational challenges they present. Drivers must continuously assess the intentions of other vehicles, decide on the appropriate lane, determine the optimal exit point, and navigate in a circular pattern. Typical driving intentions in such settings include entering, exiting, continuing within the roundabout, and lane-changing. For our simulation study, we used the *rounD* [40] dataset, which comprises natural vehicle trajectory data captured by drones over a German roundabout. Notably, this specific location lacks lane markings, making the lane hazard factor inapplicable. Given the characteristics of this dataset and the particular traffic scenario it represents, we distilled driving intentions into two primary categories: continuing within the roundabout (CR) and exiting the roundabout (ER). The input variables for this scenario are shown in Table 8.

The process of recognizing driving intentions in roundabout scenarios using TSWHMM is consistent with that in highways. Upon receiving the observation sequence O, we compute the corresponding $\tilde{P}\left(O|\lambda \right)$ based on the trained intention models ${\tilde{\lambda }}_{\mathrm{CR}}$ and ${\tilde{\lambda }}_{\mathrm{ER}}$. The driving intention is then determined by the model with the higher likelihood value. To facilitate the demonstration of recognition effects in combination with trajectories, we normalize $\tilde{P}\left(O|{\tilde{\lambda }}_{\mathrm{CR}}\right)$ and $\tilde{P}\left(O|{\tilde{\lambda }}_{\mathrm{ER}}\right)$ in the following way:

(15) $$\begin{array}{c} p_{i}=\frac{\tilde{P}\left(O|{\tilde{\lambda }}_{i}\right)}{\tilde{P}\left(O|{\tilde{\lambda }}_{\mathrm{CR}}\right)+\tilde{P}\left(O|{\tilde{\lambda }}_{\mathrm{ER}}\right)},\;i=\mathrm{CR},\mathrm{ER} \end{array}$$

(16) $$\begin{array}{c} L_{i}=\frac{e^{p_{i}}}{e^{p_{\mathrm{CR}}}+e^{p_{\mathrm{ER}}}}.\;i=\mathrm{CR},\mathrm{ER} \end{array}$$

**Figure 14:**
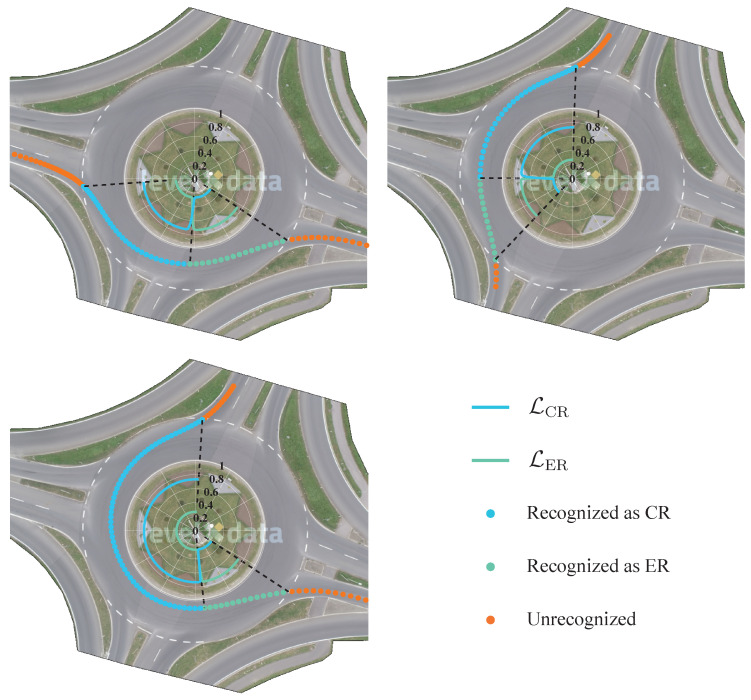
Trajectory-level recognition results in roundabout scenarios.

Figure 14 provides a detailed depiction of the model’s recognition performance for the two driving intentions. As is evident from the figure, the recognition process is remarkably stable, without frequently switching between the two intentions. Importantly, the model is capable of accurately recognizing the ER intention at an early stage, a feature that is particularly crucial for ADAS systems in roundabout scenarios. Table 9 further contrasts the performance of TSWHMM with that of HMM in the roundabout context. Specifically, for the CR intention recognition, TSWHMM achieved an accuracy of 88.8%, which is a 1.4 percentage point improvement over HMM’s 87.4%. Meanwhile, for the ER intention recognition, TSWHMM registered an accuracy of 85.8%, surpassing HMM’s 85% by 0.8 percentage points. In roundabout scenarios, the definition of TIA is similar to that of highways—it refers to how much time in advance the model recognizes the vehicle’s ER intention. And on the TIA metric, TSWHMM is 0.15 s higher compared to HMM. Given the more complex traffic environment of roundabouts, it is understandable that the improvement in TSWHMM over HMM is not as pronounced as in highway scenarios. These results underscore that, not only in highway scenarios but also in roundabout contexts, the TSWHMM with the incorporated discount factor γ effectively improves the shortcomings of HMM in sequence weight distribution.

## 6. Conclusions

To enhance the performance of the perception module in ADAS, we present the TSWHMM, a model distinguished by its superior driving intention recognition capabilities. Building upon the structure of the HMM, TSWHMM incorporates a discount factor to adjust the weights of time series data. This modification diminishes the influence of data points further from the current moment while emphasizing those closer and more indicative of the driving intention. Such an approach effectively addresses the HMM’s suboptimal weight distribution for time series data in intention recognition. Notably, as TSWHMM is an extension of the HMM and can also employ the forward algorithm to compute the observation sequence probability, it can be easily extended to other models based on the HMM, representing one of its primary advantages. In terms of model input selection, we opted for easily accessible vehicle states, such as lateral speed and heading angle. Additionally, recognizing the influence of the traffic environment on driving intentions, we introduce lane hazard factors to depict the environmental context of target vehicles. These factors aim to evaluate the hazard level surrounding the target vehicle, assisting the intention recognition model in discerning, and even predicting, the vehicle’s driving intention. Model training and testing were conducted using the *HighD* dataset. The results indicate that TSWHMM’s recognition accuracy for left and right lane changes surpasses both HMM and RNN, registering at 94.9% and 93.4%, respectively. Due to the discount factor emphasizing recent observation data, TSWHMM can recognize lane-changing intentions 0.3 s earlier than HMM. In the roundabout scenario based on the *rounD* dataset, TSWHMM consistently exhibits stable recognition capabilities. Compared to HMM, TSWHMM achieves superior accuracy for both continuing within and exiting the roundabout intentions and discerns driving intentions earlier. This further underscores its effectiveness in addressing the sequence weight distribution challenges inherent in traditional HMM, even in complex roundabout scenarios.

Given that the specific value of the discount factor has a significant impact on TSWHMM, and the optimal value of the discount factor varies in different scenarios, future improvements should focus on efficiently searching for the optimal value of the discount factor and expanding testing to include more scenarios.

## Figures and Tables

**Table 1:** Observed variables based on the *HighD*.

Symbol	Definition	Unit
Δy	Offset between the center of vehicle and the center of lane.	m
$v_{y}$	Lateral speed of vehicle.	m/s
$a_{y}$	Lateral acceleration of vehicle.	m/$s^{2}$
θ	Heading angle of vehicle.	rad
$\rho_{\mathrm{leftlane}}$	Lane hazard factor for left lane.	-
$\rho_{\mathrm{rightlane}}$	Lane hazard factor for right lane.	-
$\rho_{\mathrm{currentlane}}$	Lane hazard factor for current lane.	-

**Table 2:** Definition of parameters in lane hazard factor formula.

Parameter	Definition
A	Vehicles within the detection range (80 m) of the sensor in the corresponding lane. The current lane considers only the vehicle in front.
*a*	Constant that limits the range of lane hazard factors. In the simulation we set it to 1.
$x_{\mathrm{other}}$	Longitudinal coordinates of other vehicle’s center in corresponding lanes.
$v_{\mathrm{other}\_x}$	Longitudinal speed of the other vehicle in the corresponding lane.
$x_{\mathrm{obe}}$	Longitudinal coordinates of the target vehicle.
$v_{\mathrm{obe}\_x}$	Longitudinal speed of the target vehicle.

**Table 3:** LC Samples.

	Number of Samples	Training Set Size	Test Set Size
LCL	4500	3600	900
LCR	4500	3600	900
LK	4500	3600	900

**Table 4:** Hyperparameters in TSWHMM.

Parameter	Definition	Range of Values
$N_{\mathrm{LCL}}$, $N_{\mathrm{LCR}}$, $N_{\mathrm{LK}}$	State number of per sub-TSWHMM	From 1 to 10
$\gamma_{\mathrm{LCL}}$, $\gamma_{\mathrm{LCR}}$, $\gamma_{\mathrm{LK}}$	Discount factor of per sub-TSWHMM	From 0.01 to 1 with 0.01 interval
$M_{\mathrm{LCL}}$, $M_{\mathrm{LCR}}$, $M_{\mathrm{LK}}$	Gaussian components number of sub-TSWHMM	1
$T_{w}$	Length of time window	50 frames, i.e., 2 s

**Table 5:** Optimal set of parameters and accuracy.

	$N_{\mathrm{LCL}}$	$N_{\mathrm{LCR}}$	$N_{\mathrm{LK}}$	$\lambda_{\mathrm{LCL}}$	$\lambda_{\mathrm{LCR}}$	$\lambda_{\mathrm{LK}}$	Accuracy	
							LCL	LCR
HMM	4	4	7	1	1	1	91.9%	89.2%
TSWHMM	4	4	7	0.93	0.93	0.93	94.9%	93.4%

**Table 6:** Test set details for accuracy and TIA metrics.

Index	Test Set Characteristic	Number		Label
		LCL	LCR	
Accuracy	Including LC phase only	900	900	TestSet1
TIA	Including LK and LC phases	500	500	TestSet2

**Table 7:** Performance of TSWHMM and RNN with $T_{w}=2$ s.

Model	Accuracy			TIA		
	LCL	LCR	Average	LCL	LCR	Average
TSWHMM	94.9%	93.4%	94.2%	4.31 s	3.89 s	4.10 s
RNN	94.5%	88.0%	91.3%	3.45 s	3.63 s	3.54 s

**Table 8:** Observed variables based on the *rounD*.

Symbol	Definition	Unit
$v_{\mathrm{lon}}$	The longitudinal velocity.	m/s
$v_{\mathrm{lat}}$	The lateral velocity.	m/s
$a_{\mathrm{lon}}$	The longitudinal acceleration.	m/$s^{2}$
$a_{\mathrm{lat}}$	The lateral acceleration.	m/$s^{2}$

**Table 9:** Performance comparison of TSWHMM and HMM in roundabout scenarios.

Model	Accuracy			TIA	$N_{\mathrm{CR}}$	$N_{\mathrm{ER}}$	γ
	CR	ER	Average				
TSWHMM	88.8%	85.8%	87.3%	2.09 s	25	24	0.99
HMM	87.4%	85.0%	86.2%	1.94 s	25	24	1

## Data Availability

All data included in this study are available upon request via contacting the corresponding author.
